# Multidimensional analysis to elucidate the possible mechanism of bone metastasis in breast cancer

**DOI:** 10.1186/s12885-023-11588-6

**Published:** 2023-12-08

**Authors:** Kang Yao, Zhu Xiaojun, Zhao Tingxiao, Liao Shiyao, Ji Lichen, Zhang Wei, Li Yanlei, Tian Jinlong, Ding Xiaoyan, Zhang Jun, Bi Qing, Lv Jun

**Affiliations:** 1 Cancer Center, Department of Orthopedics, Affliated People`s Hospital, Zhejiang Provincial People`s Hospital, Hangzhou Medical College, Hangzhou, Zhejiang China; 2 Department of Laboratory Medicine, Affliated People`s Hospital, Zhejiang Provincial People’s Hospital, Hangzhou Medical College, Hangzhou, Zhejiang China; 3 Department of Musculoskeletal Oncology, Sun Yat-sen University Cancer Center, Guangzhou, Guangdong China; 4 Collaborative innovation Center for Cancer Medicine, Guangzhou, Guangdong China; 5 State Key laboratory of Oncology in South China, Guangzhou, Guangdong China; 6 Zhejiang Provincial People`s Hospital Bijie Hospital, Bijie, China

**Keywords:** Breast cancer, Bone metastasis, Prognosis, CAFs, MMP13

## Abstract

**Background:**

Breast cancer (BC) patients tend to suffer from distant metastasis, especially bone metastasis.

**Methods:**

All the analysis based on open-accessed data was performed in R software, dependent on multiple algorithms and packages. The RNA levels of specific genes were detected using quantitative Real-time PCR as a method of detecting the RNA levels. To assess the ability of BC cells to proliferate, we utilized the CCK8 test, colony formation, and the 5-Ethynyl-20-deoxyuridine assay. BC cells were evaluated for invasion and migration by using Transwell assays and wound healing assays.

**Results:**

In our study, we identified the molecules involved in BC bone metastasis based on the data from multiple BC cohorts. Then, we comprehensively investigated the effect pattern and underlying biological role of these molecules. We found that in the identified molecules, the EMP1, ACKR3, ITGA10, MMP13, COL11A1, and THY1 were significantly correlated with patient prognosis and mainly expressed in CAFs. Therefore, we explored the CAFs in the BC microenvironment. Results showed that CAFs could activate multiple carcinogenic pathways and most of these pathways play an important role in cancer metastasis. Meanwhile, we noticed the interaction between CAFs and malignant, endothelial, and M2 macrophage cells. Moreover, we found that CAFs could induce the remodeling of the BC microenvironment and promote the malignant behavior of BC cells. Then, we identified MMP13 for further analysis. It was found that MMP13 can enhance the malignant phenotype of BC cells. Meanwhile, biological enrichment and immune infiltration analysis were conducted to present the effect pattern of MMP13 in BC.

**Conclusions:**

Our result can improve the understanding of researchers on the underlying mechanisms of BC bone metastasis.

## Introduction

It is estimated that the mortality-to-incidence ratio for breast cancer (BC) is 15%, making it the most aggressive cancer among females [1]. In 2018, there were 268,670 new cases worldwide, and BC occurs in 12% of all American women over their lifetime [2, 3]. Molecular and histological evidence indicates that BC can be divided into 3 subtypes: BC expressing human epidermal receptor 2 (HER2+), hormone receptor-positive (ER+) or progesterone receptor positive (PR+) BC, and triple-negative BC (ER-, PR-, HER2-) [4, 5]. Molecularly, the therapeutic strategies for each type of BC differ, for example, hormone therapy, anti-HER2 monoclonal antibodies, and chemotherapy [6–8]. Early diagnosis and treatment have contributed to a 38% decline in BC mortality, but high-quality prevention and effective targeted treatment can accelerate the decrease [9, 10]. Genetic predisposition or family history is often associated with BC, so genomic alteration analysis could provide the most significant prognostic biomarker.

There are about 75% of stage IV BC patients who have developed skeletal metastases as the incidence of BC increases, representing about 60-70% of all metastatic BC cases [11–13]. Patients with bone metastases had a 40-month median OS in a study involving 7064 BC patients, and another study found a median survival of 50 months for BC patients with bone metastases [14, 15]. In addition to affecting survival time, bone metastasis has also negatively affected the quality of life because of fatigue, pain, and skeletal-related events (SREs) [16–18]. Treatments such as bone-modifying agents are available, but their reliability and effects on survival status limit their use [19]. To understand the underlying mechanism, we need to develop a deep understanding of the crosstalk among BC cells, fibrocytes, endothelial cells, and muscle tissue in the tumor microenvironment and bone microenvironment [20–22]. A good prognosis for BC patients with bone metastases requires suitable bone-targeting agents as well as identifying the responses of these patients through molecular mechanisms.

In our study, we identified the molecules involved in BC bone metastasis based on the data from multiple BC cohorts. Then, we comprehensively investigated the effect pattern and underlying biological role of these molecules. We found that in the identified molecules, the EMP1, ACKR3, ITGA10, MMP13, COL11A1, and THY1 were significantly correlated with patient prognosis and mainly expressed in CAFs. Therefore, we explored the CAFs in the BC microenvironment. Results showed that CAFs could activate multiple carcinogenic pathways and most of these pathways play an important role in cancer metastasis. Meanwhile, we noticed the interaction between CAFs and malignant, endothelial, and M2 macrophage cells. Moreover, we found that CAFs could induce the remodeling of the BC microenvironment and promote the malignant behavior of BC cells. Then, we identified MMP13 for further analysis. BC cells were shown to exhibit an enhanced malignant phenotype following treatment with MMP13. Meanwhile, MMP13 in BC was analyzed based on biological enrichment and immune infiltration.

## Methods

### Acquisition of open-accessed data of BC patients

Gene Expression Omnibus (GEO) and Cancer Genome Atlas Program (TCGA) databases were used for the expression profile and clinical information. Detailed, the initial expression profile file was the “STAR-Counts” form and was sorted out using the R software. Clinical information was in the bcr-xml form and sorted out using the Perl code. For the GEO database, the projects GSE14017, GSE14018 and GSE137842 were finally selected, which contain the transcriptional profiling data BC bone metastasis. The GSE16554 and GSE175692 were removed from the study because of concerns about the quality of their data. Before analysis, all the data were pro-precessed, including probe annotation, data normalization and missing value completion. With the set threshold, the limma package was used to analyze differentially expressed genes (DEGs).

### Protein-protein interaction (PPI) network

Based on input genes, we identified the PPI network. The Organism was “Homo”. All the edges and nodes were based on “evidence”. The export file generated from the STRING database was visualized using the Cytoscape software. Cytoscape’s clueGO plug-in was used to visualize enrichment analysis. The terms with P < 0.001 were identified. The hub nodes were identified using the Cytohubba plug-in.

### Prognosis analysis

As part of the prognostic analysis, univariate Cox regressions, LASSO regressions, and multivariate Cox regressions were examined. Univariate Cox Regression Analyses were employed as a preliminary step to discern the unadjusted association between each predictor variable and the survival outcome. After this, we incorporated the LASSO Regression Analysis, which particularly serves to handle high-dimensional data and is instrumental in mitigating overfitting by constraining the size of the coefficients and thus, selectively shrinking some of them to zero. Multivariate Cox Regression Analyses facilitated us to assess the mutual impact of all variables on survival, considering the possible confounding effects.

### Single-cell analysis

In our research, we extensively employed the TISCH (Tumor Immune Single-cell Hub) project for our single-cell analysis to delve deeper into the intricate cellular milieu of the tumor microenvironment. Specifically, the TISCH project, accessible via http://tisch.comp-genomics.org/home/, is a pivotal resource that provides a comprehensive and curated repository of single-cell RNA sequencing (scRNA-seq) data, predominantly focusing on the tumor immune microenvironment across diverse cancer types.

### Tumor microenvironment quantification

In our study, we employed the XCell algorithm, which serves as a valuable tool for performing cell type enrichment analysis from gene expression data for immune and stroma cell types. The XCell algorithm leverages a gene signature-based method to deduce the cellular composition of the tumor microenvironment, providing an insightful view into the complex cellular interplay within the tumor and the surrounding microenvironment [23]. We also implemented single-sample Gene Set Enrichment Analysis (ssGSEA), an extension of GSEA, which calculates separate enrichment scores for each pairing of a sample and gene set. Every sample is considered independently and, unlike the traditional GSEA, ssGSEA does not require phenotypic labels. In our work, ssGSEA was utilized to calculate enrichment scores for specified immune functions, allowing us to extract meaningful biological information regarding the active biological processes and pathways in our BC samples [24].

### Biological enrichment analysis

To elaborate, our investigation into biological enrichment commenced with the GSEA algorithm. Employing the GSEA algorithm, we conducted a focused examination of our gene expression data, leveraging specific pathway files as referenced, to identify patterns of gene expression that could be correlated with phenotypic differences, thereby revealing underlying biological processes or pathways that may be actively involved in the condition under study [25]. In addition, we executed Gene Ontology (GO) and Kyoto Encyclopedia of Genes and Genomes (KEGG) analyses utilizing the clusterprofiler package [26].

### Immunohistochemistry (IHC)

The representative image of IHC was obtained from the Human Protein Atlas (HPA) database [27]. The HPA, a comprehensive and freely accessible resource, is instrumental in providing a vast array of high-resolution IHC images, delineating protein expression patterns across various tissues and organs in the human body.

### Pan-cancer analysis

The pan-cancer analysis was performed using the online website Sangerbox (http://vip.sangerbox.com/).

### Cell culture and quantitative real-time PCR (qRT-PCR)

The human BC cell lines (T47D, MCF-7, MDA-MB-231, MDA-MB-469) and normal cell lines (MCF-10A) were routinely stored in the laboratory. All the cells were incubated in conventional culture conditions. An RNA simple Total RNA Kit (Tiangen, China) was used for total RNA, which was then reverse transcribed to cDNA. The primers used for qRT-PCR were as follows: MMP13, forward primer, 5’-ACTGAGAGGCTCCGAGAAATG-3’; reverse primer, 5’-GAACCCCGCATCTTGGCTT-3’. GAPDH, forward primer, 5’-GGAGCGAGATCCCTCCAAAAT-3’, reverse primer, 5’-GGCTGTTGTCATACTTCTCATGG-3’.

### Cell transfection

Cell transfection was executed with the aid of the Lipofectamine 2000 reagent, adhering closely to the provided protocol [28]. For ensuring the reproducibility and consistency of our experimental results, we followed a standardized transfection procedure. Before transfection, cells were seeded to ensure 70–90% confluency at the time of transfection, optimizing the cellular uptake of the plasmids. The control and sh-MMP13 plasmids, procured from Shanghai GenePharma, were then prepared at an appropriate concentration. The Lipofectamine 2000 reagent was diligently mixed with the respective plasmids, followed by an incubation period to allow the formation of Lipofectamine-plasmid complexes. Subsequently, the resultant complexes were added to the cells, facilitating the internalization of the plasmids and enabling the genetic manipulation of our target cells. After this, the transfection medium was replaced with a fresh culture medium to sustain optimal cell health and facilitate gene expression from the introduced plasmids.

### Cell proliferation assay

The evaluation of cell proliferation ability was performed using the CCK8 and colony formation assay following the standard process [28]. For the CCK8 assay, cells were seeded into 96-well plates at a predetermined density and allowed to adhere and stabilize. Post stabilization, cells were treated as per the experimental conditions, and the CCK8 reagent was added at various time points to assess proliferation rates over time. The reagent was then allowed to incubate with the cells for a specified duration, post which, the absorbance was measured at 450 nm, with the recorded values being indicative of the relative number of viable cells. Simultaneously, the colony formation assay was employed to assess the long-term proliferative potential of the cells, and thus, their ability to sustain growth over extended periods. Cells were seeded at a low density into 6-well plates to enable the observation of individual colonies and were maintained under standard culture conditions for a duration sufficient to allow colony formation. Following the incubation period, colonies were fixed, stained, and subsequently counted, with the number of colonies serving as an indicator of the proliferative capacity of the cells.

### Transwell

Following the standard protocol, we performed transwell assays with sh-MMP13 and control cells [28]. Detailed, cells undergoing study were subjected to serum starvation to synchronize. The upper chamber of the transwell insert, featuring an 8-µm pore size, was seeded with a specific number of the aforementioned cells suspended in serum-free medium while the lower chamber was filled with a medium supplemented with serum (20%), serving as a chemoattractant to drive cellular migration and invasion through the membrane. In the invasion assays, the membrane was pre-coated with Matrigel to create an additional barrier that only invasive cells could traverse, thereby distinguishing between mere migratory and invasive capabilities.

### Wound-healing

Wound-healing assay was conducted based on the sh-MMP13 and control cell**s** following the standard process [28]. The assay was utilized to evaluate the migratory capabilities of sh-MMP13 and control cells under in vitro conditions. Initially, cells were cultured in plates until they reached near confluency to facilitate the creation of a “wound” that is devoid of cells. Once appropriate confluency was achieved, a sterile pipette tip was employed to create a linear scratch, effectively generating a cell-free zone, or “wound”, within the monolayer of cells. After the creation of this wound, detached cells and debris were gently washed away, and the cells were then incubated in serum-free media to reduce proliferation and thus, isolate the effects attributable to cell migration during the healing of the wound. The progression of cell migration into the wound was monitored and documented at 0 and 24 h using microscopy.

### 5-Ethynyl-20-deoxyuridine (EdU) assay

EdU assay was performed using the sh-MMP13 and control cells following the standard process [29]. Initially, cells were seeded onto appropriate well plates and allowed to attach and proliferate under optimal culture conditions. Upon reaching the desired confluency, the cells were treated with EdU, a thymidine analog, for a specified duration, permitting its incorporation during the DNA synthesis phase of active cell proliferation. Following the incubation with EdU, the cells were fixed to preserve their structural integrity and permeabilized to allow access to the DNA. Subsequently, a copper-catalyzed click reaction was conducted wherein a fluorescently labeled azide was introduced, forming a covalent bond with the ethynyl group of EdU. Ensuing fluorescence microscopy imaging, the resultant data were quantitatively analyzed.

### Statistical analysis

The analysis based on open-accessed data was conducted using the R software. The GraphPad Prism 8 and SPSS software.

## Results

### Identification of the molecules involved in the bone metastasis process

**Fig. 1:**
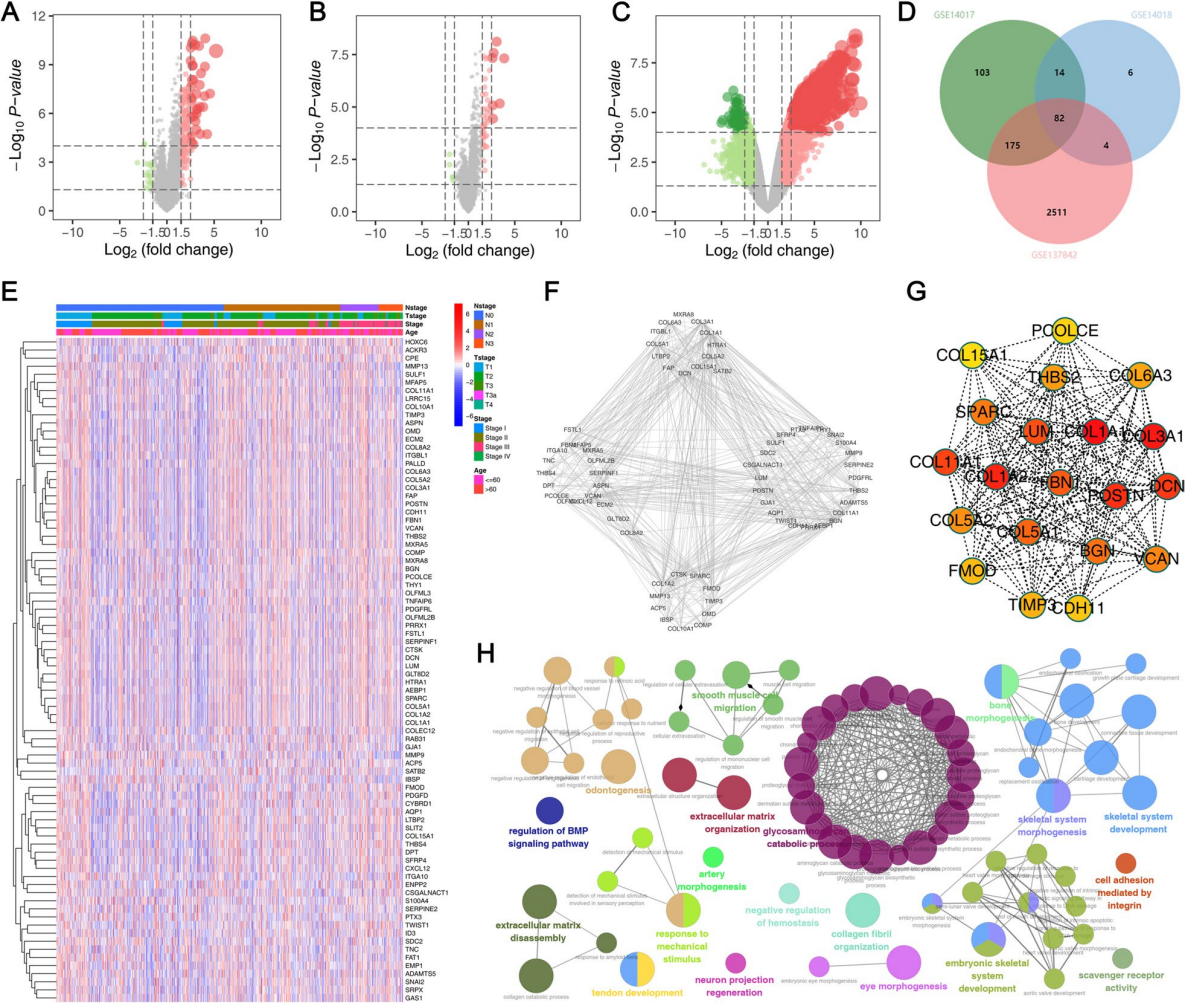
Identification of the molecules involved in the BC bone metastasis. **A** DEGs between the BC samples with metastasis of bone and other organs in GSE14017; **B** DEGs between the BC samples with metastasis of bone and other organs in GSE14015; **C** DEGs between the BC samples with metastasis of bone and other organs in GSE137842; **D** The intersection of these three databases identified 82 common genes involved in the bone metastasis process; **E** Expression pattern of these 82 common genes in BC samples; **F** PPI network based on the 82 common genes; **G** Cytohubba plug-in was utilized to identify important nodes; **H** ClueGO analysis

Based on the limma package and set threshold, we performed DEGs analysis in GSE14017, GSE14018, and GSE137842. For GSE14017, between BC samples with bone and other organ metastases, 73 genes were downregulated and 374 were upregulated (Fig. 1A). For GSE14018, in BC samples with metastases of bone and other organs, 106 genes were upregulated and 25 downregulated (Fig. 1B). For GSE137842, a total of 3189 were downregulated and 2772 upregulated genes were identified between the BC samples with metastasis of bone and other organs (Fig. 1C). The intersection of these three databases identified 82 common genes involved in the bone metastasis process (Fig. 1D). In Figs. 1E and 82 genes in the BC sample are shown to be expressed. Based on the STRING database, the PPI network was shown in Fig. 1F. The cytohubba plug-in identified that in the PPI network, the COL1A1, COL3A1, COL11A1, COL1A2, POSTN and DCN were the hub nodes, which might exert a wide regulatory effect in the BC bone metastasis (Fig. 1G). ClueGO analysis indicated that these genes were mainly enriched in the regulation of the BMP signaling pathway, odontogenesis, extracellular matrix disassembly, tendon development, glycosaminoglycan catabolic process, skeletal system development, skeletal system morphogenesis, embryonic skeletal system development (Fig. 1E).

### The bone metastasis-related genes EMP1, ACKR3, ITGA10, MMP13, COL11A1, THY1 were significanly correlated with prognosis

**Fig. 2:**
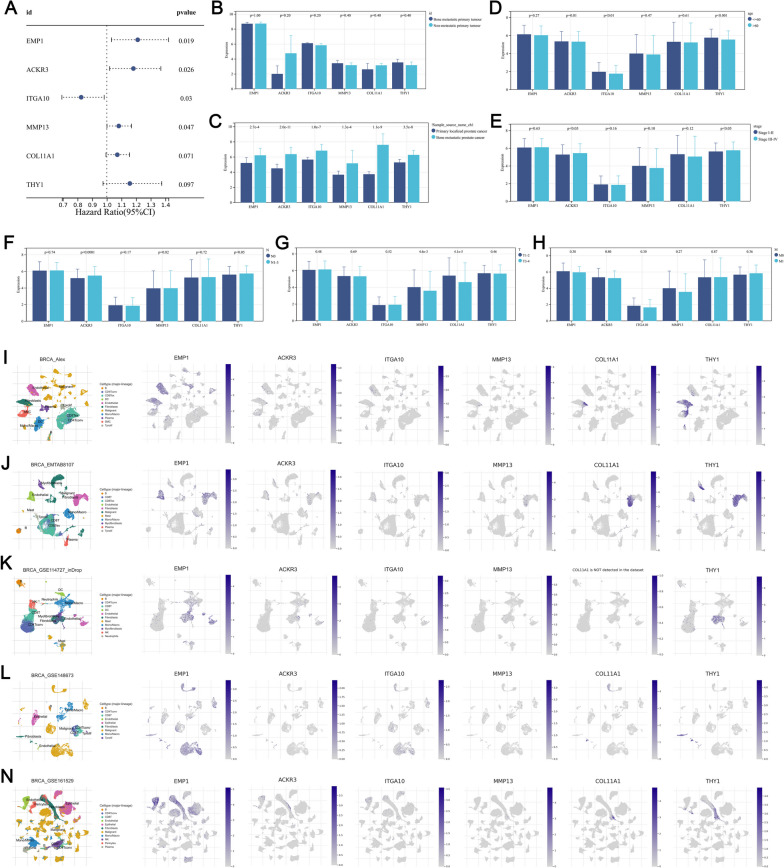
Role of bone metastasis-related genes in BC. **A** Univariate cox regression analysis identified that EMP1, ACKR3, ITGA10, MMP13, COL11A1, and THY1 were significantly correlated with patients survival; **B**-**H** Expression level of EMP1, ACKR3, ITGA10, MMP13, COL11A1, and THY1 in patients with different clinical features; **I**-**N** Single-cell analysis of EMP1, ACKR3, ITGA10, MMP13, COL11A1, and THY1 in BC samples

Univariate Cox regression analysis indicated that the EMP1, ACKR3, ITGA10, MMP13, COL11A1, and THY1 were significantly correlated with patient survival, in which the EMP1, ACKR3, MMP13, COL11A1, THY1 were risk factors, while ITGA10 was protective factor (Fig. 2A). Meanwhile, we found that all these prognosis-related genes were not differentially expressed in the primary tumor with or without bone metastasis, indicating that these genes exert their role inside the metastatic foci (Fig. 2B). Also, no remarkable difference in the level of prognosis-related genes was found in patients with < = 60 and > 60 old (Fig. 2C). Moreover, we noticed that all these genes were upregulated in the bone metastasis foci (Fig. 2D). For BC patients with stage III-IV, we noticed a higher expression level of ACKR3 and THY1 compared to those with stage I-II (Fig. 2E); For BC patients with N1-3, we noticed a higher expression level of ACKR3 and THY1 compared to those with N0 (Fig. 2F); For BC patients with T3-4, we noticed a lower expression level of MMP13 and COL11A1 compared to those with T1-2 (Fig. 2G); No significant difference was observed in the M stage (Fig. 2H). Then, we evaluated the expression pattern of these genes at the single-cell level through the TISCH website. Results indicated that these genes were primarily expressed in fibroblasts based on the BRCA_Alex, BRCA_EMTAB_8107, BRCA_GSE114727_inDrop, BRCA_GSE148673 and BRCA_GSE161529 projects (Fig. 2I).

### Cell enrichment and interaction of fibroblasts

**Fig. 3:**
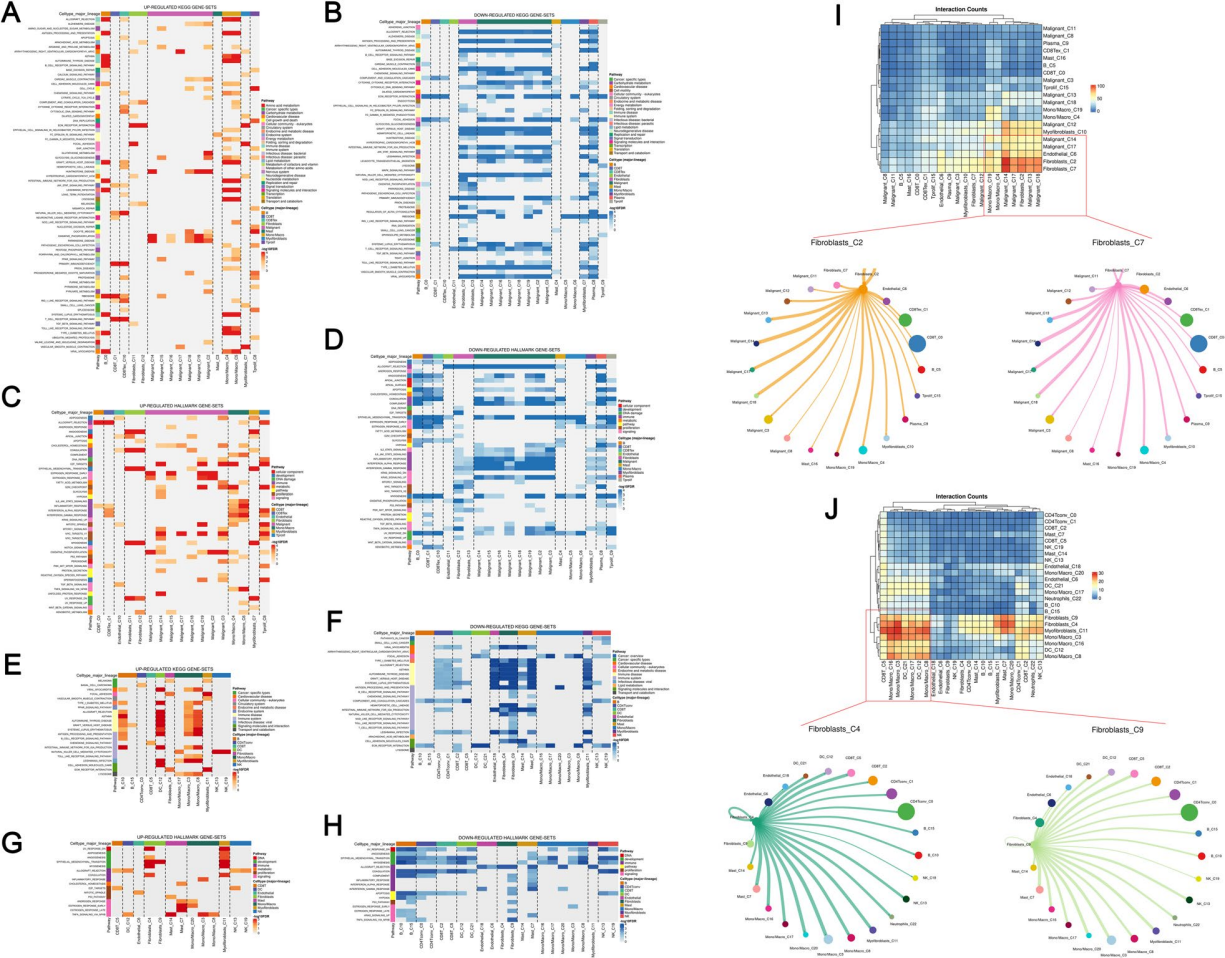
Role of CAFs in the BC microenvironment. **A** Upregulated KEGG terms in EMTAB8107 project; **B** Downregulated KEGG terms in EMTAB8107 project; **C** Upregulated Hallmark terms in EMTAB8107 project; **D** Downregulated Hallmark terms in EMTAB8107 project; **E** Upregulated KEGG terms in GSE114727_inDrop project; **F** Downregulated KEGG terms in GSE114727_inDrop project; **G** Upregulated Hallmark terms in GSE114727_inDrop project; **H** Downregulated Hallmark terms in GSE114727_inDrop project; **I** Interaction between CAFs and other cells in EMTAB8107 project; **J** Interaction between CAFs and other cells in GSE114727_inDrop project

Subsequently, the microenvironment of BC tumors is influenced by cancer-associated fibroblasts (CAFs). In the EMTAB8107 project, KEGG analysis indicated that the CAFs could activate the activity of complement and coagulation cascades and ECM receptor interaction signaling while hampering the activity of allograft rejection, antigen processing and presentation, autoimmune thyroid disease, cell adhesion molecules cams, cytosolic DNA signaling pathway, graft versus host disease, hematopoietic cell lineage, natural killer cell-mediated cytotoxicity, primary immunodeficiency, and ribosome (Fig. 3A-B). For Hallmark analysis, we noticed that CAFs could activate the activity of angiogenesis, coagulation, epithelial-mesenchymal transition (EMT), myogenesis, UV response DN, while suppressing the activity of inflammatory response, interferon alpha response, interferon-gamma response, KRAS signaling, MYC target, oxidative phosphorylation (Fig. 3C-D). Another project, GSE114727_inDrop, also showed similar conclusions (Fig. 3E-H). In the EMTAB8107 project, cell interaction results indicated that the CAFs in BC microenvironment mainly interacted with the malignant and mono/macrophage cells (Fig. 3I). In the GSE114727_inDrop project, results indicated that the CAFs primarily interacted with mono/macrophage cells (Fig. 3J).

### CAFs can remodel the Tumor microenvironment of BC

**Fig. 4:**
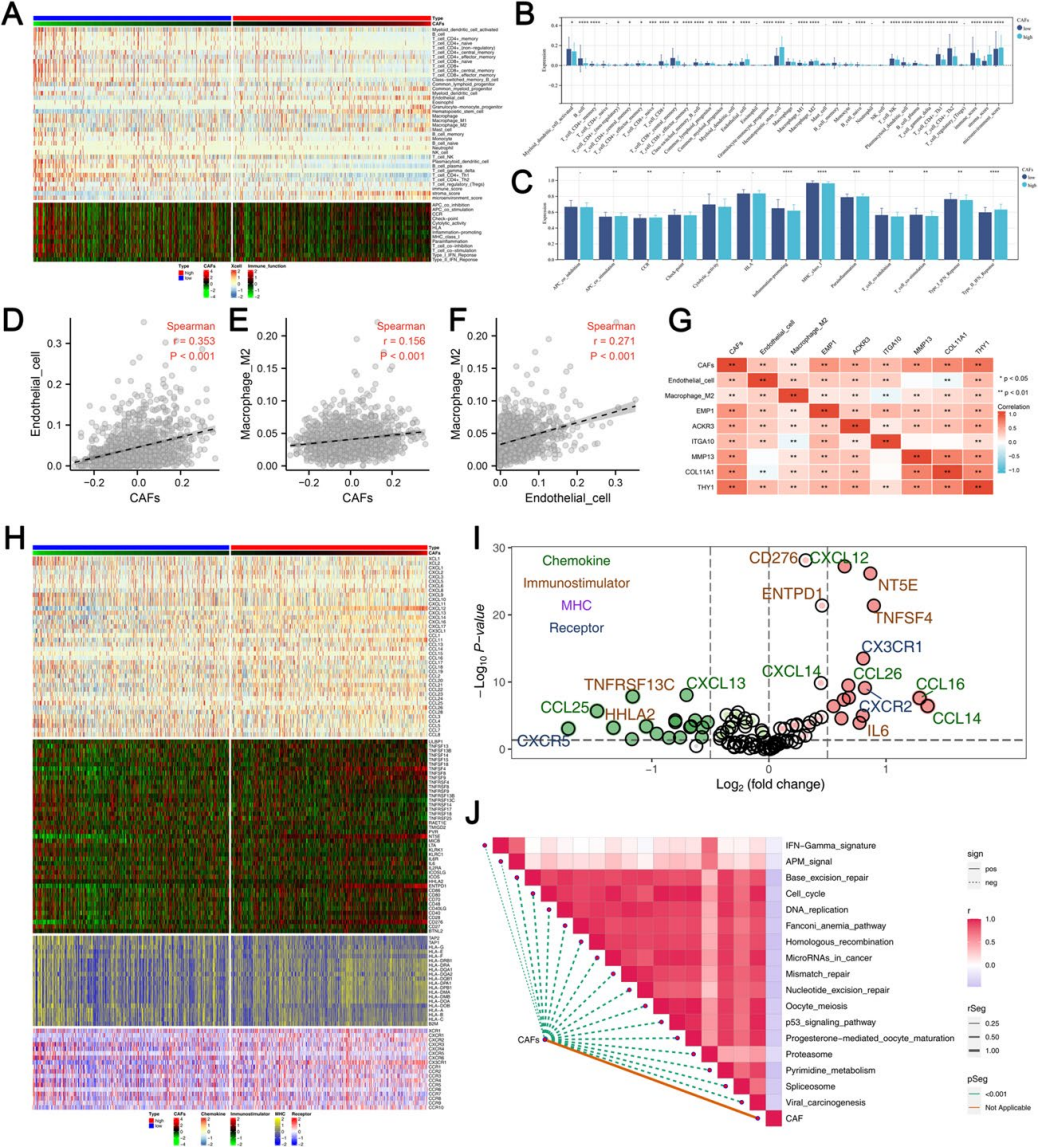
CAFs can remodel BC tumor microenvironment. **A** XCell algorithm was used to quantify the BC microenvironment; **B** The quantified immune and stromal cells in patients with high and low CAFs infiltration; **C** Quantified immune function in patients with high and low CAFs infiltration; **D** Correlation between CAFs and endothelial cells; **E** Correlation between CAFs and M2 macrophages; **F** Correlation between M2 macrophages and endothelial cells; **G** Correlation of CAFs, M2 macrophages, endothelial cells with EMP1, ACKR3, ITGA10, MMP13, COL11A1, and THY1; **H** Expression pattern of immune-related genes in BC samples; **I** The immune-related genes in patients with high and low CAFs infiltration; **J**: Pathway enrichment analysis

XCell algorithm was utilized to quantify the tumor microenvironment of BC patients (Fig. 4A). Results showed that most of the immune or stromal cells quantified by the XCell algorithm were differentially expressed in patients with different CAFs infiltration level (Fig. 4B). For the immune function quantified by the ssGSEA algorithm, we noticed that in the patients with high CAFs infiltration, the APC_co_stimulation, CCR, cytolytic activity, inflammation-promoting, MHC_class_I, parainflammation and Type_II_IFN_response were activated (Fig. 4C). Also, there is a positive correlation between the two of CAFs, endothelial cells and M2 macrophages (Fig. 4D-F, CAFs-endothelial cells: cor = 0.353, CAFs-M2 macrophages: cor = 0.156, Endothelial-M2 macrophages: cor = 0.271). The correlation of CAFs, endothelial cells and M2 macrophages with EMP1, ACKR3, ITGA10, MMP13, COL11A1, THY1 was shown in Fig. 4G. Then, we extracted the gene expression pattern of four kinds of immune molecules, including chemokine, immunostimulator, MHC and receptors (Fig. 4H). Most of these immune-related molecules were differentially expressed in patients with high and low CAFs infiltration (Fig. 4I). Almost all immune pathways were negatively correlated with CAFs, according to pathway enrichment analysis (Fig. 4J).

### BC proliferation, invasion, migration, and migration are promoted by CAFs

**Fig. 5:**
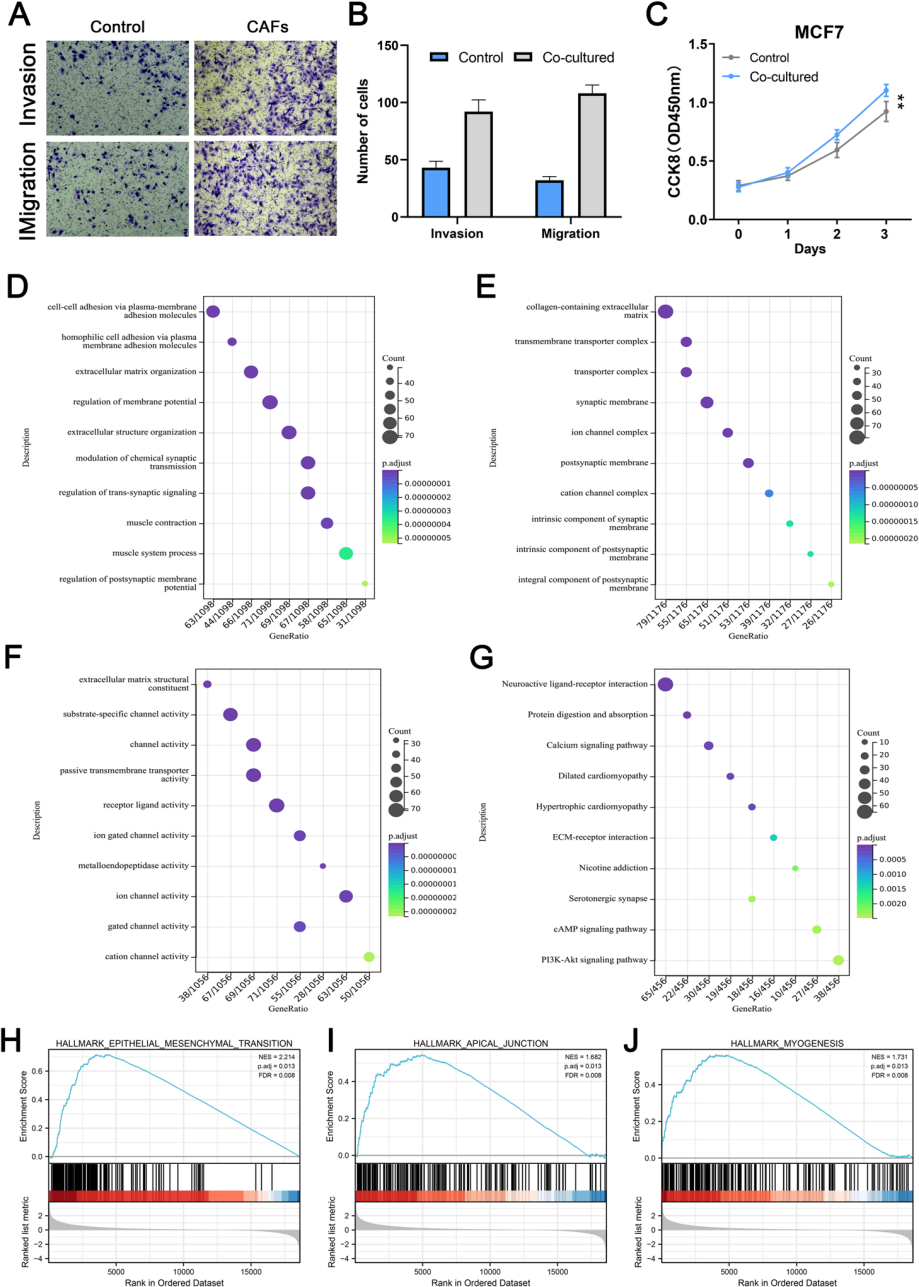
Effect of CAFs on BC cells.  **A**-**B **Transwell assay indicated the effect of CAFs on BC invasion and migration; **C** CCK8 assay indicated the effect of CAFs on BC proliferation; **D** GO-BP analysis of CAFs in BC; **E** GO-CC analysis of CAFs in BC; **F** GO-MF analysis of CAFs in BC; **G** KEGG analysis of CAFs in BC; **H**-**J** Hallmark pathways of CAFs in BC

By adding the culture supernatant of CAFs to BC cell lines, we tried to observe whether CAFs would affect the biological behavior of BC. Based on the evidence from the transwell and CCK8 assay, the results of the transwell assay indicated that CAFs could promote the invasion, migration and proliferation ability (Fig. 5A-C). Results of GO-BP indicated that CAFs significantly affect the activity of cell-cell adhesion via plasma-membrane adhesion molecules, homophilic cell adhesion via plasma membrane adhesion molecules, extracellular matrix organization, and regulation of membrane potential (Fig. 5D). Results of GO-CC showed that CAFs was remarkably correlated with the collagen-containing extracellular matrix, transmembrane transported complex, transporter complex, and synaptic membrane (Fig. 5E). Results of GO-MF indicated that the CAFs were mainly enriched in the term of extracellular matrix structural constituent (Fig. 5F). Results of KEGG were also shown in Fig. 5G. The top three enriched Hallmark pathways of CAFs were EMT, apical junction and myogenesis (Fig. 5H-J).

### Further exploration of MMP13 in BC

**Fig. 6:**
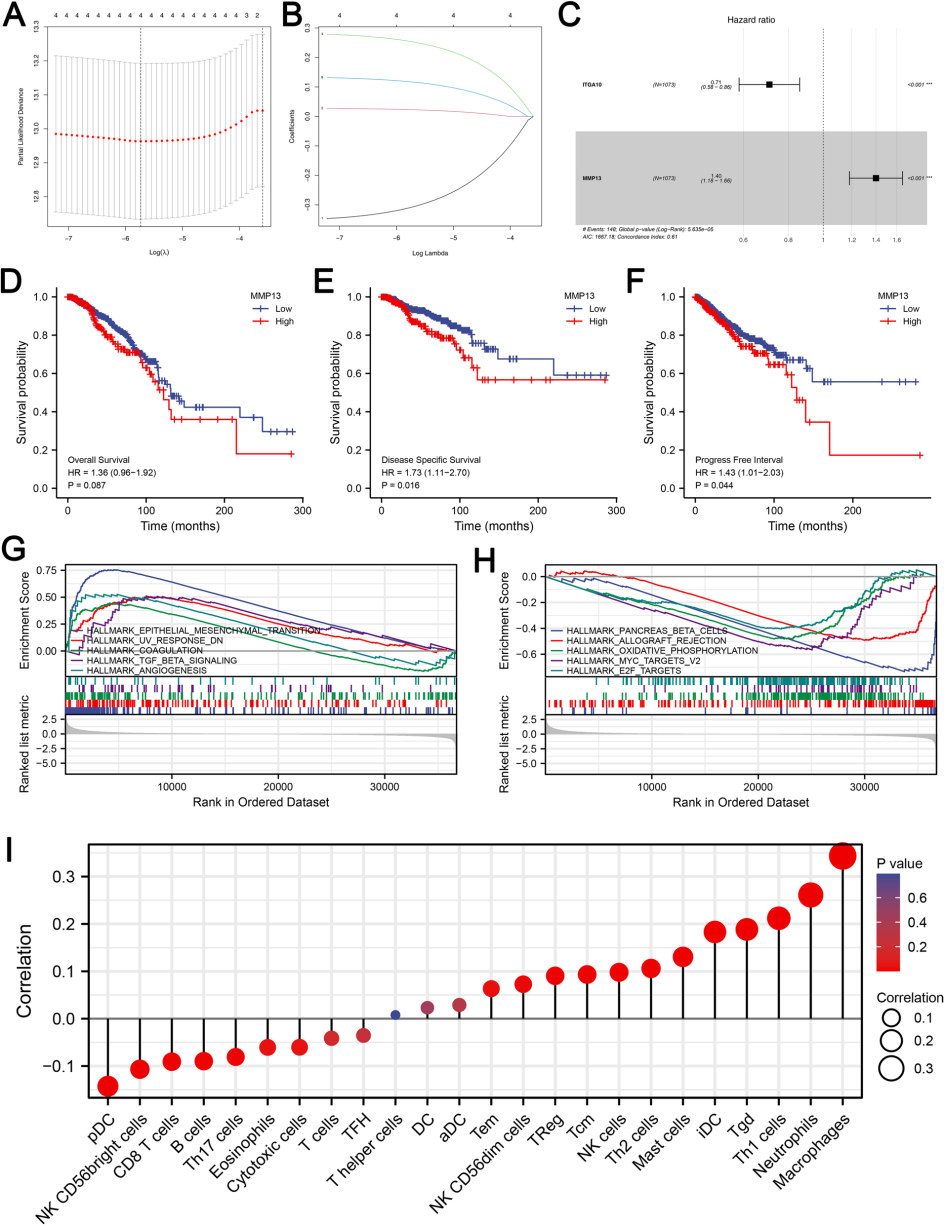
Further exploration of MMP13. **A**-**B** LASSO regression analysis; **C** Multivariate Cox regression; **D**-**F** Prognosis analysis of MMP13; **G** The top five upregulated Hallmark pathways of MMP13; **H** The top five downregulated Hallmark pathways of MMP13; **I** Immune infiltration analysis of MMP13

As a next step, we performed LASSO regression analysis to reduce the number of dimensions in the data (Fig. 6A-B). Then we identified two genes, including ITGA10 and MMP13 based on the molecules screened (Fig. 6C). The MMP13 was then selected for further analysis. There is a poor prognosis for patients with high MMP13 expression, whether it is overall survival, disease-free survival or progression-free survival (Fig. 6D-F). Hallmark analysis showed that in patients with high MMP13 expression, the top five upregulated pathways were EMT, UV response DN, coagulation, TGF-β signaling, and angiogenesis (Fig. 6G). The top downregulated pathways were pancreas beta cells, allograft rejection, oxidative phosphorylation, MYC target and E2F targets (Fig. 6H). Based on the results of the immune infiltration analysis, MMP13 corresponds negatively to macrophages, neutrophils, Th1 cells, TgD and iDC, while corresponds negatively to pDC, NK CD56 bright cells and CD8 + T cells (Fig. 6I).

### Biochemical effects of MMP13 on BC cells

**Fig. 7:**
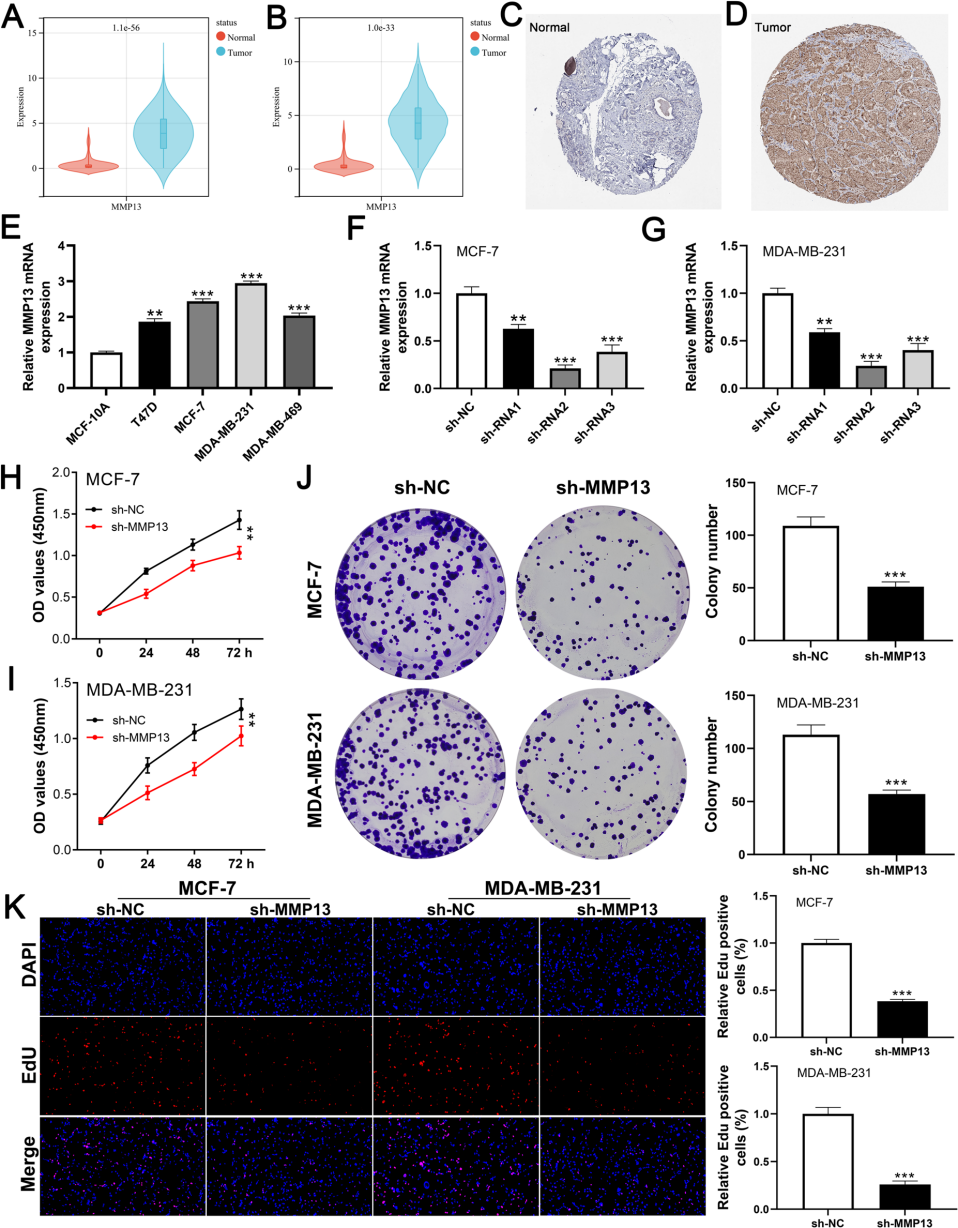
MMP13 promotes BC cell proliferation.  **A**-**B** The expression level of MMP13 in BC and control normal tissue; **C**-**D** Representative IHC image of MMP13 in BC and control normal tissue; **E** The expression level of MMP13 in BC cell lines; **F**-**G** The knockdown efficiency of MMP13; **H**-**I** CCK8 assay in sh-MMP13 and control cells; **J** Colony formation assay in sh-MMP13 and control cells; **K** EdU assay in sh-MMP13 and control cells

**Fig. 8:**
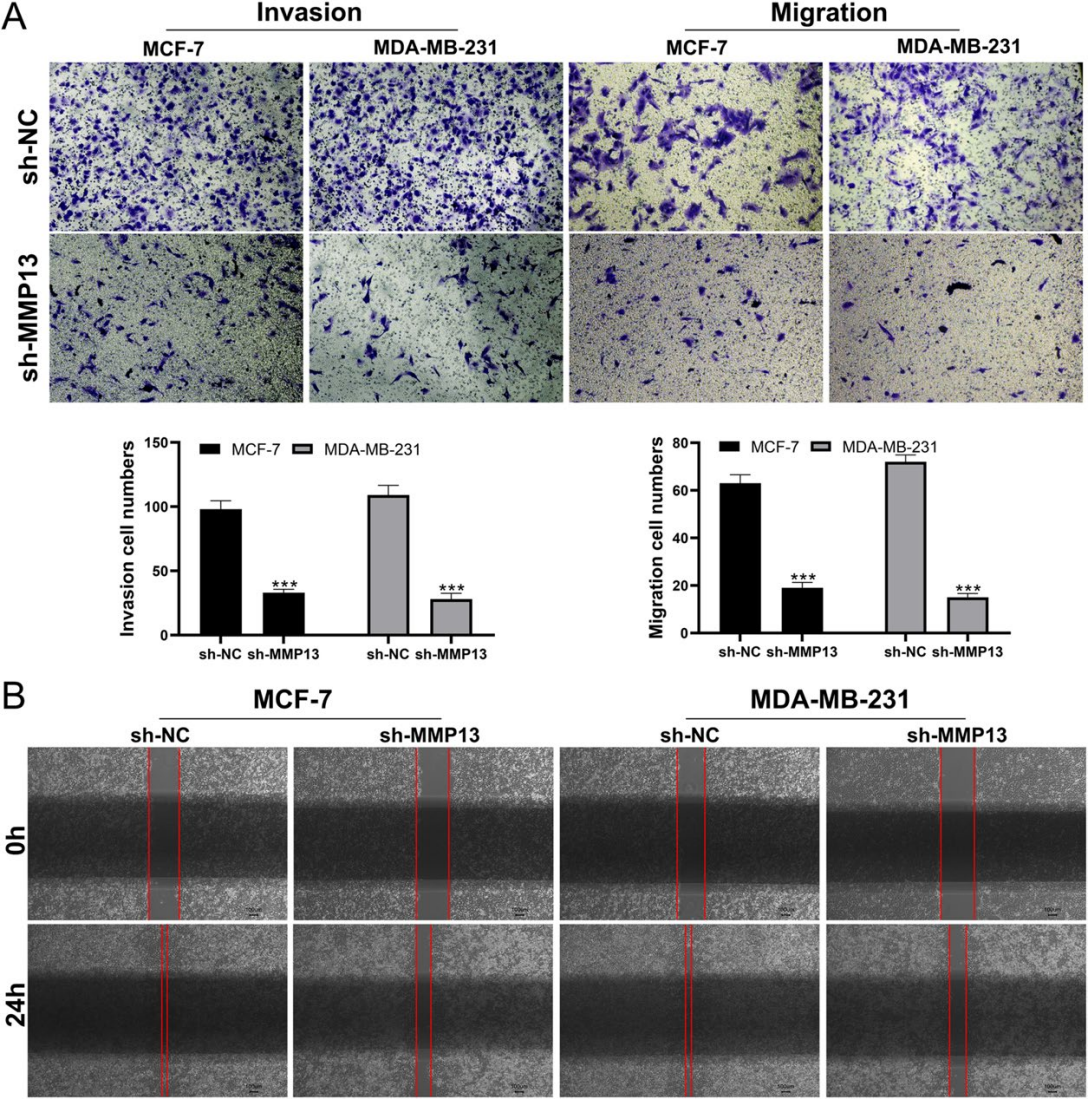
MMP13 promotes the invasion and migration of BC cells. **A** Transwell assay in sh-MMP13 and control cells; **B** Wound healing assay in sh-MMP13 and control cells

We noticed a higher level of MMP13 in paired or non-paired BC tissue compared to the control normal tissue (Fig. 7A-B). Also, the IHC result was obtained from the HPA database and we found the protein level of MMP13 is higher in BC samples (Fig. 7C-D). Cell lines expressing MMP13 were found to be highly expressed (Fig. 7E). qRT-PCR was used to validate knockdown efficiency and shRNA#2 was selected for further analysis. (Fig. 7F-G). As shown by CCK8 and colony formation assays, the inhibition of MMP13 significantly impeded the proliferation of BC cells. (Fig. 7H-J). This effect was also validated by the EdU assay (Fig. 7K). BC cell invasion and migration were significantly inhibited by suppressing MMP13 in transwell and wound-healing assays. (Fig. 8A-B).

### Pan-cancer analysis of MMP13

**Fig. 9:**
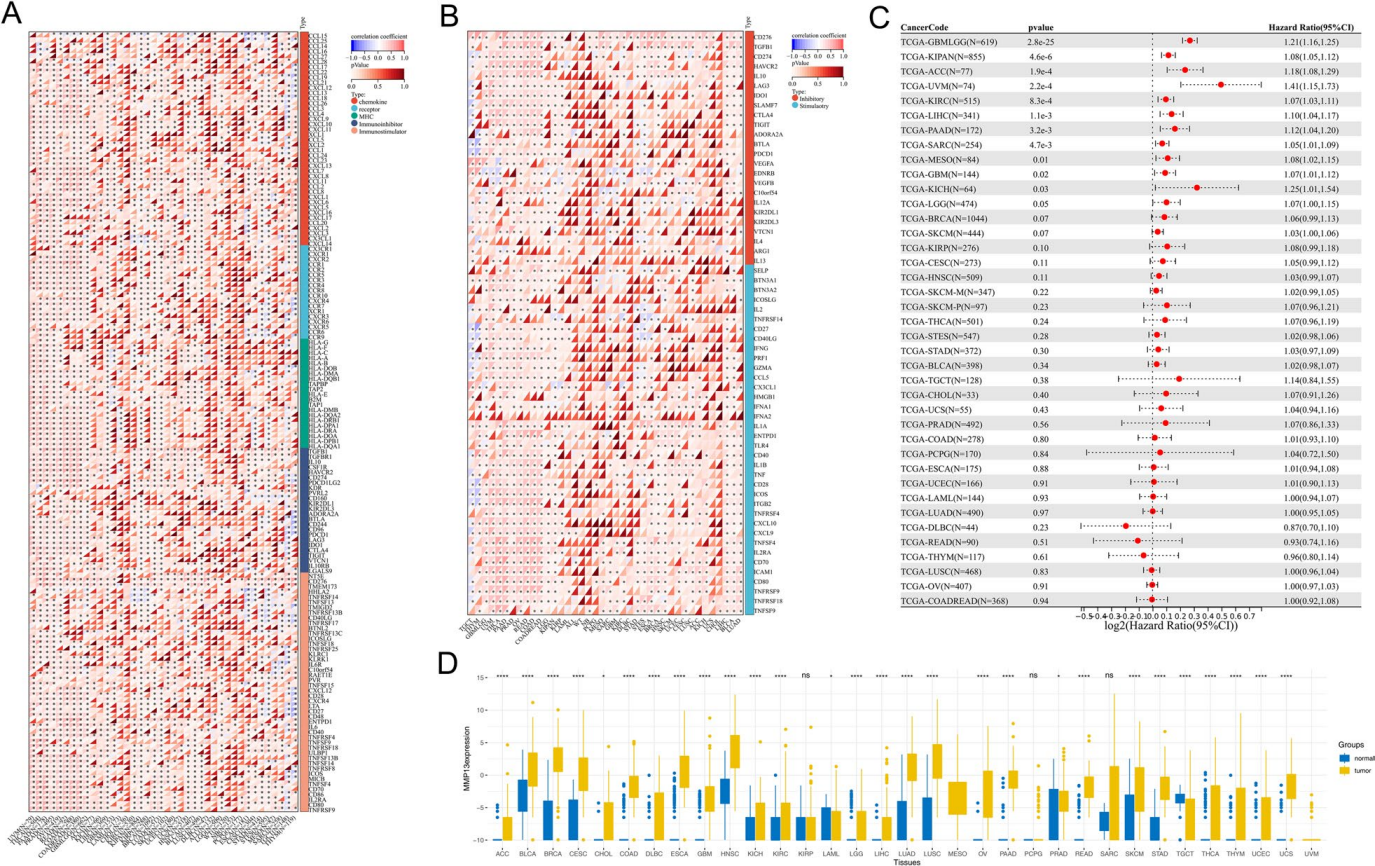
Pan-cancer analysis of MMP13. **A** Correlation between the MMP13 and immune-related genes in pan-cancer; **B** Correlation between the MMP13 and immune checkpoint in pan-cancer; **C** Prognosis analysis of MMP13 in pan-cancer; **D** Expression level of MMP13 in pan-cancer

We next evaluated the effect pattern of MMP13 in pan-cancer. A significant correlation was found between MMP13 and immune-related molecules in pan-cancer, including chemokine, immunostimulator, MHC and receptor (Fig. 9A). Moreover, MMP13 was correlated with many immune checkpoints in pan-cancer (Fig. 9B). The prognosis effect and expression pattern of MMP13 in pan-cancer was shown in Fig. 9C-D.

## Discussion

The BC morbidity rate continues to increase steadily and has become a major public health concern, accounting for 25% of cancer incidence and 15% of cancer-related deaths in women [1]. Although mammographic screenings are still helping diagnose BC, the current prevention and treatment strategies are ineffective [30]. A genetic risk factor, including pathogenic mutations in genes such as checkpoint kinase 2, considerably impacts BC development; inherited mutations in BRCA1 and BRCA2 account for the majority of cases [31]. There are no underlying mechanisms that are capable of preventing or treating BC effectively. In light of mutations of genes, the microenvironment of tumors has been altered, and we can enhance the immune system’s role in the fight against cancer to benefit patients. The horizon of BC treatment has been continuously expanding, with immunotherapy emerging as a groundbreaking advancement [32]. In other cancer types, notably melanoma and non-small-cell lung cancer, immunotherapeutic approaches, including immune checkpoint inhibitors (such as anti-PD-1 and anti-CTLA-4 antibodies) [33], have demonstrated remarkable success in improving patient outcomes [34, 35]. For BC, especially in cases expressing PD-L1, immunotherapy has been explored and applied, showcasing promising results particularly in combination with chemotherapy, thus offering a beacon of hope for translating the success observed in other cancers to the realm of breast cancer treatment [36]. The genetic landscape, especially mutations in BRCA1 and BRCA2, plays a pivotal role in BC development by disrupting the normal tumor-suppressive functions, thereby propelling unchecked cellular proliferation and genomic instability [37]. Individuals harboring these mutations often confront an escalated risk of developing breast and other cancers at a relatively younger age [38]. Recognizing these genetic predispositions, through genetic testing, allows for tailored risk-management strategies, offering preventive and prophylactic interventions, and shaping personalized treatment approaches. Therefore, it is important to find new targets for BC.

BC patients with bone metastases have low survival rates, with less than 30% surviving over 5 years [39]. The majority of BC metastases occur in the bones, except for the liver, brain, and lung. It is defined as the dissemination of cancer cells from primary cancer to secondary sites, which is the evolution of BC metastasis to bone [40]. When cancer cells enter the bone marrow, they destroy bone tissue by interacting with osteoblasts, osteoclasts and bone stromal cells, and the cancer-associated stromal fibroblasts release various growth factors stored in bone tissue, causing cancer cells to proliferate continuously to form metastases [41]. The function of vascular endothelial cells, hematopoietic stem cells, osteoblasts, osteoclasts, fibroblasts and immune cells is critical during the process of metastasis and the control of tumor dormancy at secondary sites [42–44].

Bone metastasis in BC profoundly imperils patient outcomes, instigating a series of skeletal-related events (SREs), including spinal cord compression and pathological fractures, which mandate interventional procedures like surgery and radiotherapy, each harboring considerable morbidity and complex, pain-laden management challenges [45]. The distinct microenvironment of the bone underscores a complex interplay among tumor cells, osteoblasts, and osteoclasts, facilitating not only tumor progression but also resistance to conventional therapies, thereby spotlighting an urgent, unmet medical need [46]. Investigating the molecular mechanics behind BC bone metastasis becomes pivotal against this backdrop, offering insights into the intricate interactions shaping both localized and systemic tumor microenvironments, while fostering tumor survival and further proliferation [47]. Our research aims to meticulously unravel these molecular dynamics, providing crucial insights into the biological processes that promote tumor cell resilience and expansion within the bone marrow niche. This exploration is imperative to identify prospective molecular and cellular therapeutic targets, comprehend mechanisms conferring resistance to established therapies, develop strategies to mitigate complications such as SREs, and refine prognostic precision. By illuminating the complex molecular pathways implicated in BC bone metastasis, we aspire to pave avenues for innovative, targeted therapies, which not only enhance management efficacy and alleviate morbidity but also elevate survival and quality of life, by providing strategic options to potentially delay or even prevent metastasis in high-risk patients, thereby enhancing patient outcomes and expanding therapeutic horizons. Bioinformatics can exert a useful tool for understanding diseases [48, 49]. Our data analysis in the study was rigorously executed using a battery of bioinformatic algorithms and tools. We employed different algorithms for analysis. The utility of bioinformatics allows us to decipher the convoluted nature of diseases like cancer, unraveling molecular pathways and identifying potential biomarkers or therapeutic targets by processing and synthesizing vast and multifaceted biological data, which are imperative for comprehending the molecular mechanisms driving the disease [50]. In our study, we identified the molecules involved in BC bone metastasis based on the data from multiple BC cohorts. Then, we comprehensively investigated the effect pattern and underlying biological role of these molecules. We found that in the identified molecules, the EMP1, ACKR3, ITGA10, MMP13, COL11A1, and THY1 were significantly correlated with patient prognosis and mainly expressed in CAFs. Therefore, we explored the CAFs in the BC microenvironment. Results showed that CAFs could activate multiple carcinogenic pathways and most of these pathways play an important role in cancer metastasis. Meanwhile, we noticed the interaction between CAFs and malignant, endothelial and M2 macrophage cells. Moreover, we found that CAFs could induce the remodeling of the BC microenvironment and promote the malignant behavior of BC cells. Then, we identified MMP13 for further analysis. The result showed that MMP13 could enhance the malignant phenotype of BC cells. Meanwhile, biological enrichment and immune infiltration analysis were conducted to present the effect pattern of MMP13 in BC.

CAFs are a heterogeneous stromal cell population and the most important component of the tumor microenvironment. Our result showed that bone metastasis-related genes were mainly expressed in CAFs. Also, in the BC microenvironment, the patients with high CAFs infiltration might have a higher activity of multiple pathways involved in cancer metastasis, including EMT, angiogenesis and coagulation. Meanwhile, CAFS can inhibit the inflammatory response. In colon cancer, Hu et al. found that exosomes secreted by CAFs could enhance chemotherapy resistance and metastasis ability by regulating EMT pathways and tumor stemness [51]. Kashima et al. showed that in esophageal cancer, CAFs could promote lymph node metastasis [52]. In BC, Wen et al. noticed that the IL32 generated by CAFs could enhance the invasion and metastasis ability through integrin β3-p38 MAPK signaling [53]. Zheng et al. found that the biglycan secreted by CAFs in BC can exert a cancer-promoting factor by inducing the immunosuppressive microenvironment [54]. Our results indicated that the CAFs might contribute to the bone metastasis process of BC, making it a potential target for clinical applications. Moreover, we found that CAFs could significantly promote the invasion, migration, and proliferation ability of BC cells.

MMP13 is a member of matrix metalloprotease. It is reported that MMP is an interesting gene associated with cancer progression, angiogenesis promotion, metastasis and immune surveillance avoidance [55]. Zhang et al. indicated that in ovarian cancer, HIF-1α could promote cancer invasion and migration by targeting MMP13 and inducing the hypoxia microenvironment [56]. Kumamoto et al. noticed that ING2 can facilitate colon cancer progression by increasing the expression level of MMP13 [57]. Also, Ma et al. found that MMP3 and MMP13 are the hub genes of anaplastic thyroid cancer based on transcriptome sequencing [58]. MMP13’s capability to degrade collagens and other ECM components enables cancer cells to navigate through the physical barriers and intravasate into the circulatory system, propelling metastatic spread [59]. Examples from literature distinctly showcase that MMP13 overexpression has been correlated with elevated invasive and metastatic potential in various cancers, such as colorectal cancer and osteosarcoma, underscoring its crucial role in modulating tumor microenvironment and influencing cancer progression [57, 60].

Although we have carried out a lot of analysis to elaborate on the possible mechanism of bone metastasis of BC and obtained some trustworthy results, some shortcomings still need to be mentioned. Firstly, underlying race bias is inevitable. Detailed, the enrolled sample was mostly Western populations. However, there are still differences in genomics among different races, which may reduce the reliability of our conclusions. Secondly, follow-up biological research is still needed to validate our conclusions.

## Data Availability

statement. Breast cancer samples were gathered from openly available databases including the TCGA database (https://tcga-data.nci.nih.gov/tcga/) and the GEO database (https://www.ncbi.nlm.nih/geo/query/). Other data that support the findings of this study are available on request from the corresponding author.
