# Correction: Multidimensional analysis to elucidate the possible mechanism of bone metastasis in breast cancer

**DOI:** 10.1186/s12885-023-11750-0

**Published:** 2023-12-21

**Authors:** Kang Yao, Zhu Xiaojun, Zhao Tingxiao, Liao Shiyao, Ji Lichen, Zhang Wei, Li Yanlei, Tian Jinlong, Ding Xiaoyan, Zhang Jun, Bi Qing, Lv Jun

**Affiliations:** 1 Cancer Center, Department of Orthopedics, Affiliated Peopl’s Hospital, Zhejiang Provincial Peopl’s Hospital, Hangzhou Medical College, Hangzhou, Zhejiang China; 2 Department of Laboratory Medicine, Affiliated Peopl’s Hospital, Zhejiang Provincial People’s Hospital, Hangzhou Medical College, Hangzhou, Zhejiang China; 3 Department of Musculoskeletal Oncology, Sun Yat-sen University Cancer Center, Guangzhou, Guangdong China; 4 Collaborative innovation Center for Cancer Medicine, Guangzhou, Guangdong China; 5 State Key laboratory of Oncology in South China, Guangzhou, Guangdong China; 6 Zhejiang Provincial Peopl’s Hospital Bijie Hospital, Bijie, China

**Correction: BMC Cancer 23, 1213 (2023)**

10.1186/s12885-023-11588-6

Following publication of the original article [1], the authors reported that the author Lv Jun, was erroneously not marked as co-corresponding author. This correction confirms that Lv Jun is indeed a corresponding author to the original article [1].

The original article [1] has been corrected.
